# Variation of Gut Microbiome in Free-Ranging Female Tibetan Macaques (*Macaca thibetana*) across Different Reproductive States

**DOI:** 10.3390/ani11010039

**Published:** 2020-12-27

**Authors:** Binghua Sun, Xiaojuan Xu, Yingna Xia, Yumei Cheng, Shuxin Mao, Xingjia Xiang, Dongpo Xia, Xi Wang, Jinhua Li

**Affiliations:** 1 School of Resource and Environmental Engineering, Anhui University, Hefei 230601, China; xiayingna1996@126.com (Y.X.); m17855131664@163.com (Y.C.); shuxinmao1011@163.com (S.M.); xjxiang@ahu.edu.cn (X.X.); wangxi198307@163.com (X.W.); 2 International Collaborative Research Center for Huangshan Biodiversity andTibetan Macaque Behavioral Ecology, Anhui University, Hefei 230601, China; dpxia@163.com; 3 School of Life Science, Hefei Normal University, Hefei 230601, China; jannehus@126.com; 4 School of Life Science, Anhui University, Hefei 230601, China

**Keywords:** gut microbiome, female reproduction, Proteobacteria, Tibetan macaque

## Abstract

**Simple Summary:**

The gut microbiome is expected to adapt to the varying energetic and nutritional pressures in females of different reproductive states. Although the genus *Macaca* has the widest geographical range of nonhuman primates, few empirical studies are currently available that explore the relationship between female reproductive states and their gut microbiome in this genus. We have examined variation of gut bacterial microbiome in free-ranging female Tibetan macaques (*Macaca thibetana*) across different reproductive states (cycling, pregnancy and lactation). We found significant changes in gut bacterial taxonomic composition, structure and their potential functions in different reproductive states of our study species. In particular, the relative abundance of Proteobacteria increased significantly during pregnancy and lactation. In addition, the relative abundance of Succinivibrionaceae and *Succinivibrio* (Succinivibrionaceae) were overrepresented in pregnant females, whereas Bifidobacteriaceae and *Bifidobacterium* (Bifidobacteriaceae) were overrepresented in lactating females. Furthermore, predicted functional genes of several metabolic pathways related to host’s energy and nutrition, such as metabolism of carbohydrates, cofactors and vitamins, glycans and other amino acids, were overrepresented in pregnancy and lactation. Thus, our results suggest that the gut microbiome may play an important role in the energetic and nutritional strategies of female reproductive ecology in the genus *Macaca.* Future studies of the “microbial reproductive ecology” of primates that incorporate food availability, reproductive seasonality, female reproductive physiology and gut inflammation are warranted.

**Abstract:**

The gut microbiome is expected to adapt to the varying energetic and nutritional pressures in females of different reproductive states. Changes in the gut microbiome may lead to varying nutrient utilizing efficiency in pregnant and lactating female primates. In this study, we examined variation in the gut bacterial community composition of wild female Tibetan macaques (*Macaca thibetana*) across different reproductive states (cycling, pregnancy and lactation). Fecal samples (n = 25) were collected from ten adult females harvested across different reproductive states. Gut microbial community composition and potential functions were assessed using 16 S rRNA gene sequences. We found significant changes in gut bacterial taxonomic composition, structure and their potential functions in different reproductive states of our study species. In particular, the relative abundance of Proteobacteria increased significantly during pregnancy and lactation. In addition, the relative abundance of Succinivibrionaceae and *Succinivibrio* (Succinivibrionaceae) were overrepresented in pregnant females, whereas Bifidobacteriaceae and *Bifidobacterium* (Bifidobacteriaceae) were overrepresented in lactating females. Furthermore, the relative abundance of predicted functional genes of several metabolic pathways related to host’s energy and nutrition, such as metabolism of carbohydrates, cofactors and vitamins, glycans and other amino acids, were enriched in pregnancy and lactation. Our findings suggest that changes in the gut microbiome may play an important role in meeting the energetic needs of pregnant and lactating Tibetan macaques. Future studies of the “microbial reproductive ecology” of primates that incorporate food availability, reproductive seasonality, female reproductive physiology and gut inflammation are warranted.

## 1. Introduction

Cycling, pregnancy and lactation are three important reproductive states for adult female mammals. Changes in these reproductive states affect female energetic and nutritional needs [1,2]. In particular, to meet the fetus and the infant’s needs for energy and nutrition, females face greater energetic and nutritional pressure during pregnancy and lactation. In terms of daily energy expenditure of a female mammal, compared with the cycling state, pregnancy increases daily energy expenditure by 20–30%, and lactation increases daily energy expenditure by 37–39% [3]. Similarly, nutrient requirements, such as vitamins, are greatly enhanced in pregnancy and lactation states, as shown in lab rats [4]. In both human and nonhuman primates (NHPs), the high dietary protein requirements during lactation increase by at least one-third [2]. Thus, reproduction is a costly endeavor for female mammals that must bear the major burden of investment in offspring [5]. How to meet the requirements of energy and nutrition during pregnant and lactating states is an important adaptive problem for female mammals, and limits their individual reproductive success.

Mammals have evolved many behavioral and physiological strategies to meet their energy and nutrient requirements during pregnant and lactating states [1,6]. These strategies include increasing feeding time, a preference for high-protein and high-calorie foods [7,8], decreasing physical activity, which serves to reduce energy expenditure [9,10], and enhancing metabolic efficiency [11], as well as using seasonal reproduction [12]. Recent studies highlight that the gut microbiome can help the host to process indigestible food resources such as plant structural carbohydrates, and then increase energy and nutrient uptake [13,14]. Changes in the gut microbiome are closely related to the energy homeostasis of the host [15], as well as the digestive efficiency and nutritional states [16]. Therefore, the gut microbiome may open new doors to understand how adult female mammals adapt to energetic and nutritional pressures in the state of reproduction.

Recent studies implied that the community composition and diversity of the gut microbiome can co-vary with different reproductive states, and these changes are beneficial to the host, so they can meet the requirements of energy and nutrition during pregnancy and lactation. For example, in pregnant woman, an overall increase in Proteobacteria and Actinobacteria could lead to an increase in metabolic efficiency, which was also associated with low gut microbial diversity and high rates of inflammation [17]. In NHPs, Mallott et al. (2020) recently found that the gut microbial diversity of Phayre’s leaf monkey (*Trachypithecus phayrei crepusculus*) was lower in pregnancy than that of other reproductive states, and reproductive hormone concentrations contribute to the gut microbial shifts during pregnancy [18]. Similarly to this human study, Proteobacteria and Actinobacteria were higher in pregnant females of white-faced capuchins (*Cebus capucinus*), but the alpha diversity of their gut microbial community remained stable across different reproductive states [6]. Moreover, studies on pigs also showed that the composition and function of their gut microbiome are closely related to host metabolism during pregnancy and lactation, as well as affecting the milk composition of lactating pigs [19,20]. Nevertheless, few empirical studies are currently available that explore the relationship between female reproduction and their gut microbiome in wild living primates, due to the difficulty of sample collection and identification of individuals and their reproductive states.

Genus *Macaca* has the widest geographical range of nonhuman primates [21]. As a species of this genus, the Tibetan macaque (*Macaca thibetana*) is endemic to east central China. The diet of this species varies seasonally and is characterized by a predominance of young leaves in the spring, mature leaves in the summer, mature leaves and fruits/nuts in the fall, and mature leaves, bark, stem and fallen nuts in the winter. The free-ranging Tibetan macaques of our study group inhabit the subtropical habitat in Anhui Province. The group named Yulinkeng 1 (YA1) has been monitored continuously since 1986. All individuals can be identified by specific physical characteristics such as body size, fur color, facial features, and body scars [22]. Gestation length of this species is about 5.5 months and usually starts in late autumn. Lactation begins in the early spring of the next year and lasts about a year. The birth and weaning days of infants are well documented by our team for long-term behavioral ecology. In this study, we use this study group to examine: (1) whether the alpha and beta diversity of the gut microbiome showed significant variation in different reproductive states of female Tibetan macaques, and (2) whether the taxonomic compositions and potential functions of the gut microbiome differ with each reproductive state.

## 2. Materials and Methods

### 2.1. Study Subject and Sample Collection

This study was carried out at the Valley of Wild Monkeys (VWM), a tourist destination located in Mt. Huangshan National Reserve. The site represents a highly seasonal ecosystem. During our study period, the YA1 group of Tibetan macaques contained 41 individuals (18 adults). This free-ranging group has been habituated and monitored for nearly 30 years, and therefore we were able to individually identify and follow each macaque at close range in order to collect fresh stool samples [23].

In the present study, the end of lactation, defined as weaning behavior, was observed. It has been reported that the average length of pregnancy in *Macaca* is 168 days (163–176 d) [24], and gestation length in Tibetan macaques is about 5.5 months. To make sure the stool sample was taken during gestation, pregnancy was defined retrospectively as ~5 months prior to birth. A cycling female was defined as a non-pregnant (~6 months prior to birth) and non-lactating (weaning behavior was observed) female. Due to the limitations of our small sample size, we were not able to distinguish different stages of lactation (early, mid, and late lactation) and pregnancy (first, second, and third trimesters of pregnancy). After filtering through the above threshold, 25 fresh fecal samples of 10 adult females were identified (Table S1). Definition of age groups: Yong adult (≥5–10 years old), Middle adult (≥10–15 years old), Old (≥15 years old) [22]. In total, the samples used throughout this study include three states of cycling (6 fecal samples), pregnancy (6 fecal samples) and lactation (13 fecal samples). Fresh feces were collected by following individuals and stored immediately with RNAlater (QIAGEN, Valencia, CA, USA). Samples were shipped to the laboratory at Anhui University and stored at −80 ℃ after reaching the lab (≤2 h).

### 2.2. DNA Extraction and Sequencing

DNA was extracted from frozen stool samples using a QIAamp DNA Stool Mini Kit (Qiagen, Inc., Valencia CA), following the manufacturer’s protocol with a bead-beating procedure. Total DNA extracted from 25 stool samples was sent to the Shanghai Majorbio Bio-pharm Technology Co., Ltd. (Shanghai, China) for analysis. For each sample, the V3–V4 region of the 16 S rRNA gene was amplified using primers 338F (5′-ACTCCTACGGGAGGCAGCAG-3′) and 806R (5′-GGACTACHVGGGTWTCTAAT-3′) as previously described [25]. Reaction conditions consisted of an initial 94 °C for 5 min, followed by 30 cycles of 94 °C for 45 s, 53 °C for 45 s, 45 s at 72 °C, and a 10 min final extension at 72 °C. PCR products were purified with a Min Elute PCR Purification Kit (QIAGEN) and then quantified using the QuantiFluor-ST and the dsDNA System (Promega, Madison, WI, USA). Purified amplicons were pooled in equal amounts, and pair-end 2 × 300 bp sequencing was performed on Illlumina Miseq platform at Shanghai Majorbio Bio-pharm Technology Co., Ltd. (Shanghai, China).

### 2.3. Sequence Analysis

Sequences of 25 samples were processed using Qiime 2 (version, 2020.2). Default parameters were used for demultiplexing. Specifically, quality control was performed with the DADA 2 function within Qiime 2 to truncate forward and reverse reads, to denoise the data and for detection and removal of chimeras. The representative sequence variants of each sample were retained and assigned to bacterial taxa using Naive Bayes classifier trained on Greengenes (version, gg_13_8). For the singletons, which are likely due to sequence errors or low-level contaminations, ASVs with less than 2 counts were filtered out.

### 2.4. Data Analysis

Alpha diversity (Shannon and ASVs richness indices) was calculated using Mothur (http://www.mothur.org/wiki/Schloss_SOP#Alpha_diversity). Beta diversity of the Non-metric Multidimensional Scaling (NMDS) and Adonis analysis were performed on the Qiime 2 (vision 2020.2). The box plot of alpha diversities and the NMDS plots of the dissimilarity metrics were visualized using OriginPro 2016. To explore the functional profiles of our bacterial community data set, the functional profiles of microbial communities were predicted using Phylogenetic Investigation of Communities by Reconstruction of Unobserved States (PICRUSt).

Linear Mixed Effects Models (LMMs) were used to detect the potential multiple influences of season, age, and reproductive states on the relative abundance of dominant phyla and alpha diversity. Individual identity was included as a random effect in the LMMs. The response variable was log-transformed or square root transformed where needed to meet the model assumptions. The same sets of predictors (age, season, reproductive state, and season*reproductive state) were included in all starting models. F-test statistics were calculated by Kenward–Roger’s approximation for degrees of freedom using the lsmeans package. We conducted post hoc analyses on all models and *p*-values were corrected for multiple comparisons using Bonferroni correction, with a significance level of 0.05. To detect the biomarkers of microbial taxa and predicted genes of KEGG pathways in different sample groups, linear discriminant analysis effect sizes (LEfSe) were assessed using a non-parametric factorial Kruskal–Wallis rank-sum test (*p*-value > 0.05; LDA threshold > 2) according to the online protocol (https://huttenhower.sph.harvard.edu/galaxy/). The above analysis did not include longitudinal comparisons of the same individuals due to the limitation of sample size.

### 2.5. Data Availability

The raw data were submitted to the Sequence Read Archive (SRA) of NCBI under the accession number PRJNA663108.

## 3. Results

### 3.1. Microbial Community Profiles

**Figure 1:**
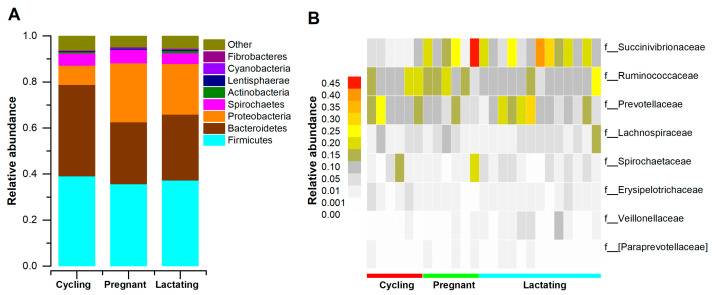
The distributions of main phyla and families in different reproductive states. (**A**): Relative abundance of gut microbial taxa at the phylum level. Stacked bar graphs illustrate the abundances of phyla, and x-axis represents the samples. (**B**): The distributions of families (the average relative of all fecal samples > 0.01). A heatmap was used to show family patterns.

After quality filtering, and flattening out according to the minimum sample sequence, we acquired 422,702 high-qualities reads with 16,908 (ranging from 10,818 to 24,557 across all 25 samples) sequences per sample. The gut microbiome of Tibetan macaques contained twelve known phyla and fifty-two known genera. The dominant bacterial phyla were Firmicutes (x = mean ± SD, x = 39.1 ± 9.9%), Bacteroidetes (x = 35.28 ± 9.5%), and Proteobacteria (x = 15.89 ± 8.6%) (Figure 1A). There were eight families with an average relative abundance greater than 1%, which was dominated by Prevotellaceae (x = 14.54 ± 7.10%), Ruminococcaceae (x = 15.04 ± 4.23%) and Succinivibrionaceae (x = 16.18 ± 11.64%) (Figure 1B). At the genus level, Tibetan macaques’ gut microbiota was dominated by *Prevotella* (x = 14.54 ± 7.10%), *Succinivibrio* (x = 16.18 ± 11.64%), and *Treponema* (x = 4.43 ± 5.08%).

### 3.2. Alpha and Beta Diversity

**Figure 2:**
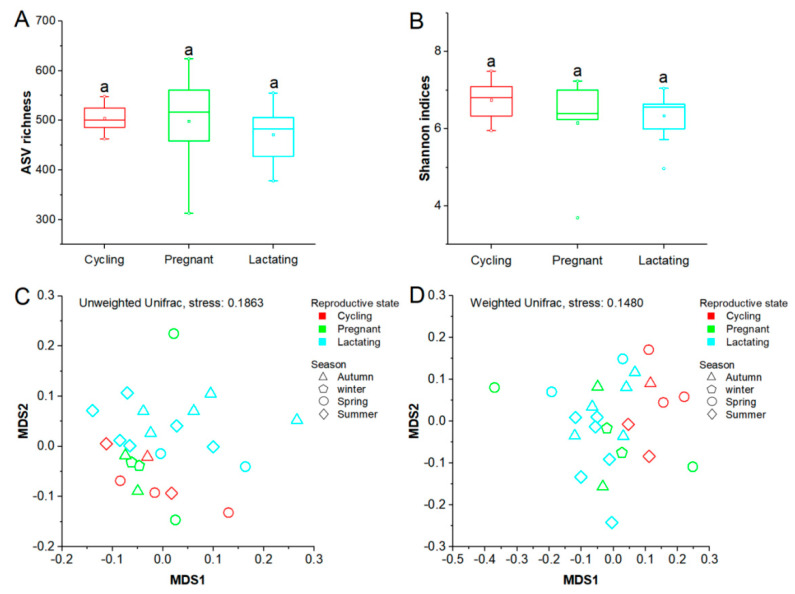
Differences in gut microbiome diversity of three reproductive states. (**A**): Comparison of ASV richness. (**B**): Comparison of Shannon diversity indexes. (**C**) and (**D**): Differentiation of gut microbiome structure. NMDS was used to show differentiation patterns of the three reproductive states. Adonis tests were performed on unweighted and weighted Unifrarc distance metrics. Significance level was 0.05.

There was no evidence that reproductive status, age, or season had a significant effect on alpha diversity of Tibetan macaques (Shannon indices, *p* > 0.05; ASVs richness indices, *p* > 0.05) (Table S2). Furthermore, we did not detect any significant difference in Shannon index or ASV richness between different reproductive states (*p* > 0.05) (Figure 2A,B). However, when NMDS and Adonis tests were performed on unweighted and weighted Unifrac dissimilarities to investigate the variations of beta diversity between reproductive states, we found that reproductive state had a significant effect on the differentiation of gut microbial community structure (unweighted, F = 1.549, R^2^ = 0.132, *p* = 0.013; weighted, F = 2.053, R^2^ = 0.157, *p* = 0.015) (Figure 2C,D). Furthermore, our results showed that there were no significant differences in the microbial community structure between any two reproductive states evaluated by the unweighted Unifrac distances (cycling/pregnant, *p* = 0.256; cycling/lactating, *p* = 0.087; pregnancy/lactating, *p* = 0.094). However, the two dyads cycling/pregnant and pregnancy/lactating were significantly different based on the weighted Unifrac distances (cycling/pregnant, *p* = 0.014; cycling/lactating, *p* = 0.003; pregnancy/lactating, *p* = 0.742) (Table S3). In addition, we observed significant differentiation of gut microbiome structure across different seasons based on unweighted Unifrac distances (unweighted, F = 1.447, R^2^ = 0.171, *p* = 0.021; weighted, F = 1.300, R^2^ = 0.157, *p* = 0.190) (Figure 2C,D), while no significant differentiation across different age groups was detected (unweighted, F = 1.441, R^2^ = 0.116, *p* = 0.055; weighted, F = 0.770, R^2^ = 0.065, *p* = 0.685) (Figure S1A,B).

In order to examine the variation of taxonomic composition across different reproductive states, we first used LMMs to assess the potential mixed effects of reproductive state, seasonal and age factors on dominant phyla (Firmicutes, Bacteroidetes and Proteobacteria) (account for 87.8% of the total) and the ratio of Firmicutes to Bacteroidetes (F/B) (Table S2). Our results showed that the phylum Proteobacteria was significantly influenced by the factor of reproductive state (df1 = 2, df2 = 12; F = 5.508, *p* = 0.020), but not significantly influenced by the seasonal and age factors (*p* > 0.05). Moreover, the phylum Bacteroidetes was significantly influenced by the factors of reproductive state (df1 = 2, df2 = 14; F = 6.155, *p* = 0.012). In contrast, we did not find that the phylum Firmicutes was significantly influenced by reproductive state, seasonal and age factors (*p* > 0.05), or the ratio of F/B (*p* > 0.05). 

**Figure 3:**
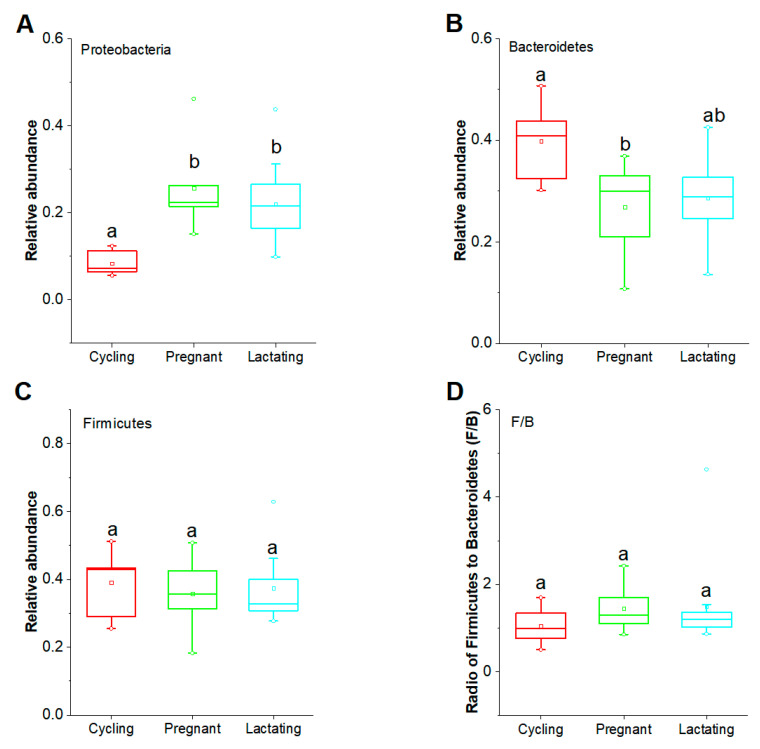
Variations of the dominant phyla across three reproductive states. (**A**): Comparison of the dominant phylum Proteobacteria. (**B**): Comparison of the dominant phylum Bacteroidetes. (**C**): Comparison of the dominant phylum Firmicutes. (**D**): Comparison of the ratio of F/B. Host hoc analyses in Linear Mixed Effects Models (LMMs) and *p*-values were corrected for multiple comparisons using the Bonferroni correction. Significance level was 0.05.

Furthermore, we found that the relative abundance of Proteobacteria was significantly lower in cycling state than in pregnant and lactating states (Cycling vs. Pregnant, *p* = 0.025; Cycling vs. Lactating, *p* = 0.025), whereas, there was no significant difference between pregnant and lactating states (*p* = 0.725) (Figure 3A). The relative abundance of Bacteroidetes was significantly higher in the cycling state than the pregnant state (Cycling vs Pregnant, *p* = 0.043; Cycling vs Lactating, *p* = 0.069), while there was no significant difference between pregnancy and lactation (*p* = 0.389) (Figure 3B). The relative abundance of Firmicutes, as well as the radio of Firmicutes to Bacteroidetes (F/B), did not differ significantly between any two reproductive states (*p* > 0.05) (Figure 3C,D). 

**Figure 4:**
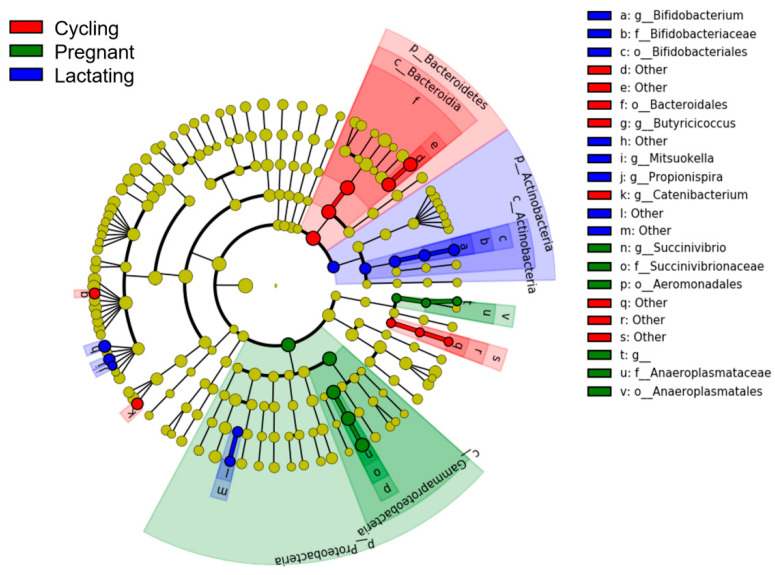
LEfSe analysis on the gut microbial taxonomy of three reproductive states. Gut microbial taxonomy enriched in different reproductive states identified by linear discriminant analysis coupled with effect size (LEfSe) (LDA > 2, *p* < 0.05).

LEfSe analyses showed that each reproductive state was characterized by different bacterial taxa (from phylum to genus) (LDA > 2, *p*-value < 0.05) (Figure 4). Three known taxa were overrepresented in cycling samples, including o_Bacteroidales, *g_Butyricicoccus* and *g_Catenibacterium*. Five known taxa, including o_Anaeroplasmatales, f_Succinivibrionaceae, f_Anaeroplasmataceae, o_Aeromonadales, and *g_Succinivibrio*, were overrepresented in pregnant samples. We also found that six known taxa were overrepresented in lactating samples, which were o_Bifidobacteriales, f_Bifidobacteriaceae, *g_Bifidobacterium*, *g_Mitsuokella*, and *g_Propionispira*.

### 3.3. Predicted Metabolic Functions

**Figure 5:**
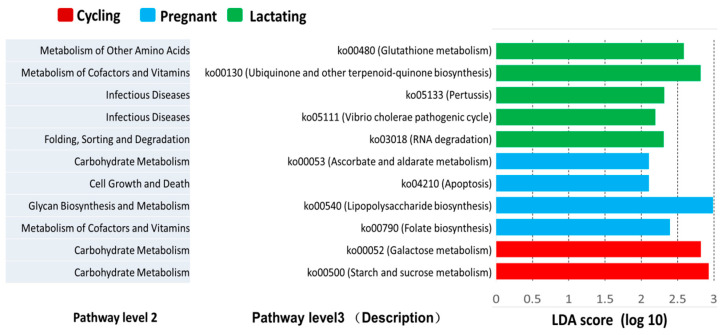
LEfSe analysis on the predicted functional genes of three reproductive states. The predicted functional genes enriched in different reproductive states identified by linear discriminant analysis coupled with effect size (LEfSe) (LDA > 2, *p* < 0.05).

Furthermore, we also found several predicted metabolic functions (KEGG pathway level 3) were enriched in one of the three reproductive states based on LEfSe results (LDA > 2, *p*-value < 0.05) (Figure 5). The predicted functional genes of four KEGG pathways ko00790 (Folate biosynthesis), ko00540 (Lipopolysaccharide biosynthesis), ko00053 (Ascorbate and aldarate metabolism), and ko04210 (Apoptosis) were overrepresented in lactating individuals. Additionally, five KEGG pathways ko03018 (RNA degradation), ko05111 (Vibrio cholerae pathogenic cycle), ko05133 (Pertussis), ko00130 (Ubiquinone and other terpenoid-quinone biosynthesis) and ko00480 (Glutathione metabolism) were overrepresented in pregnant individuals. Moreover, two KEGG pathways ko00500 (Starch and sucrose metabolism) and ko00052 (Galactose metabolism) were overrepresented in cycling individuals.

## 4. Discussion

We found that the reproductive state of wild living female Tibetan macaques was significantly related to gut microbial community structure (evaluated by beta diversity). This is consistent with previous studies of the gut microbiome in NHPs, pigs and mice [6,18,26,27]. However, we detected an overall stability of the alpha diversity of different reproductive states (pregnant, lactating and cycling), which is consistent with the result of wild living white-faced capuchins studied by Mallott and Amato (2018) [6]. In contrast, shifts in gut microbial alpha diversity during pregnancy and lactation have been reported in a number of vertebrates [18,28]. For example, alpha diversity of the gut microbiome increased in pregnancy and lactation of bats [28], while pregnancy reduced alpha diversity in human, wild Phayre’s leaf monkeys, captive mice and oviparous lizards [17,18,29,30]. These results, together with our current findings from wild Tibetan macaques, suggested that the gut microbial diversity of different reproductive states may be influenced by complex factors. Previous studies have reported that female hormones, diet and immune responses can co-vary with different reproductive states [31,32,33], which are strongly associated with host gut microbial diversity and composition [17,18,34,35]. To explain why changes in the diversity of gut microbiome of different study subjects were so varied, future studies of reproduction-associated changes in the gut microbiome diversity that incorporate data on reproductive hormones, host diet and gut inflammation are warranted.

In addition to differences in fecal mycobiota diversity, previous studies have shown that changes in gut microbial composition are related to the energy requirements of pregnant and lactating states; for example, Proteobacteria and Actinobacteria increased in pregnancy, while Bacteroidetes decreased in pregnancy and lactation [17,26]. Here, we found that the relative abundance of Bacteroidetes was significantly higher in the cycling state. Given that several strains of Bacteroidetes are beneficial to the host immune system [36,37], the reduction of this phylum indicated an immune function decrease in pregnancy of Tibetan macaques. In addition, we did not find a significant increase of Actinobacteria during the pregnant state. This finding has also been observed in humans and wild living white-faced capuchins [6,17]. In particular, we found that the relative abundance of Proteobacteria increased significantly during pregnancy and lactation of female Tibetan macaques, similar to that reported in humans and NHPs [6,17,38], indicating an important role of this phylum in female reproduction. In fact, previous studies have demonstrated that Proteobacteria is associated with energy accumulation [15,17,38]. For instance, increased relative abundance of Proteobacteria is beneficial to Tibetan macaques and lab mice accumulate energy in response to the energy demand of a cold environment [15,39]. The underlying mechanisms resulting in the alteration of the microbial communities remain to be clarified, but we speculate that the relative abundance of Proteobacteria increased in female Tibetan macaques during pregnancy and lactation may facilitate energy uptake during reproduction and meet their energy needs.

However, the role of Proteobacteria in the mammalian gut remains unclear. Previous studies have shown that several pathogenic bacteria, belonging to Proteobacteria, are enriched in pregnant females [16,40]. For instance, the potentially pathogenic genera *Stenotrophomonas* and *Roseomonas* were characterized in pregnant females of wild white-faced capuchins [6]. Additionally, an increased relative abundance of Proteobacteria has also been associated with low gut microbial diversity and high rates of inflammation in humans and mice [15,17,41]. Here, we found that pregnant females of Tibetan macaques were characterized by *Succinivibrio* enrichment. As a genus of Proteobacteria, *Succinivibrio* has been reported to be normally enriched in ruminal and Hadza hunter-gatherers’ gut microbiomes [42,43]. Moreover, the diversity of the gut microbiome of Tibetan macaques did not decrease as the relative abundances of Proteobacteria increased during pregnancy and lactation. Based on the available data, we do not know whether the increased relative abundance of Proteobacteria also was associated with high rates of gut inflammation in the macaques during pregnancy and lactation. It will be of great interest to investigate the relationship between gut microbiome and gut inflammation in Tibetan macaques in a future study, which will help us clarify the role of Proteobacteria in the adult female gut.

Notably, it has been observed that the family Lachnospiraceae (Firmicutes) was abundant in pregnant females of white-faced capuchins [6]. A similar result was also detected during the first trimester in pregnant women [17]. Instead, we found that the dominant family Succinivibrionaceae (Proteobacteria) was significantly enriched in pregnant female Tibetan macaques. The same is true of the dominant genus *Succinivibrio* (Succinivibrionaceae) (mean relative abundance of all samples was 16.18%). Succinivibrionaceae and *Succinivibrio* are usually enriched in ruminal microbiota [42]. Members belonging to these taxa were efficient in fermenting glucose through the production of acetic and succinic acids [44], as well as in aiding in the metabolism of different types of fatty acids [45]. Moreover, the genus *Succinivibrio* increased significantly during the seasons of diet-related energy shortfalls in Tibetan macaques and Hazda hunter gatherers of Tanzania [39,43]. Thus, previous evidence implied that Succinivibrionaceae and *Succinivibrio* might help female Tibetan macaques cope with energy accumulation during pregnancy. We also found that the genus of *Bifidobacterium* (Bifidobacteriaceae of phylum Actinobacteria) was overrepresented in lactating females of Tibetan macaques. As important probiotics, members of *Bifidobacterium* could produce short-chain fatty acids (SCFAs) including lactic, acetic, propionic, and butyric acid [46], which may benefit the lactating Tibetan macaques’ health, such as biological barrier, nutrition, immune enhancement and improvement of gut function [47,48,49].

Additionally, based on the predicted metagenome data, we observed changes in the relative abundance of functional genes in different reproductive states involved in several metabolic pathways. These pathways are mainly related to host energy and nutrition, including carbohydrate metabolism, cofactor and vitamin metabolism, glycan biosynthesis and metabolism, and metabolism of other amino acids. This finding was distinct from what has been found in humans and white-faced capuchins [6,17], whereas, it signals an important role of the gut microbiome in female Tibetan macaques for reproductive energy and nutrition. For example, increased folate biosynthesis (belonging to cofactor and vitamin metabolism pathways) is likely related to increased nutrient needs during lactation [50]. However, the mean weighted nearest-sequenced taxon index (NSTI) for our samples was 0.160 (SD = 0.029). The accuracy of predictions is similar to previously reported analyses in several mammals (mean NSTI = 0.140) and Tibetan macaques (mean NSTI = 0.142), but lower than for humans (mean NSTI = 0.03) [39,51]. Thus, our data describing potential functional differences in the gut microbiome between reproductive states of Tibetan macaques should be interpreted cautiously. In this regard, to evaluate the functional role of the gut microbiome in wild female Tibetan macaques, additional studies based on metagenomic sequencing are warranted.

Finally, previous studies revealed that mammals have evolved many behavioral and physiological strategies to meet energy and nutrient requirements during pregnancy and lactation. Female hormones, diet and immune responses can co-vary with different reproductive states [31,32,33]. These factors are associated with host gut microbial diversity and composition [17,18,34,35]. Our study group lives in a seasonal environment, and reproduction of these macaques is highly seasonal [23]. We also found that seasonal changes could affect the female gut microbiome in this study. Thus, it is possible that changes in food availability or reproductive seasonality, or both, influence the shifts in the gut microbial communities across different reproductive states. Despite our results indicating that changes in the gut microbiome may play an important role in meeting the energetic needs of Tibetan macaques during pregnancy and lactation, the small sample size as well as the limitations of our understanding make it difficult to fully interpret the above results. These limitations, involving effects of other potential factors of the gut microbiome in wild Tibetan macaques, remain unknown, including female diet, reproductive physiology, immune responses and environmental microbiome. In addition, the gut microbiome may vary in different stages of pregnancy (early, mid, and late lactation). For instance, in humans, the composition and function of the gut microbiome vary significantly at different stages of pregnancy [17]. The question of whether the gut microbiome of female Tibetan macaques changes in different stages of pregnancy remains to be studied. Furthermore, sample collection of the gut is difficult and sometimes forbidden, particularly for protected wildlife. Therefore, the gut microbiome is widely studied by fecal sampling, even if the fecal microbiome does not represent the real gut microbiome directly. A recent study on captive rhesus macaques (*Macaca mulatta*) showed that fecal microbiota were highly representative of the colonic lumen and mucosa, which supported the feasibility of using fecal samples to study the gut micriobiome of primates [52], at least in the genus *Macaca*.

## 5. Conclusions

Our findings provided evidence of significant changes in composition, structure and potential function of the gut microbiome in different reproductive states (pregnant, lactating and cycling) of female Tibetan macaques. In particular, the dominant family Succinivibrionaceae (Proteobacteria) and abundant genus *Succinivibrio* (Succinivibrionaceae) were overrepresented in pregnant females of Tibetan macaques, whereas the genus *Bifidobacterium* (Bifidobacteriaceae) was overrepresented in lactating females. Furthermore, the relative abundance of predicted functional genes of several metabolic pathways related to host’s energy and nutrition were overrepresented in pregnant and lactating female Tibetan macaques. Our results imply that changes in the gut microbiome in different reproductive states of females may play an important role in the reproduction of female Tibetan macaques. However, the small sample size as well as the limitations of our understanding of the role of other potential and important factors, including food availability, reproductive seasonality, female reproductive physiology and gut inflammation, in the gut microbiome of female Tibetan macaques during different reproductive states limit our results. Future studies of the “microbial reproductive ecology” of primates that incorporate these potential factors, as well as using the method of metagenomics sequencing, will help to elucidate how the gut microbiome contributes to the energetic and nutritional strategies of female primates.

## Data Availability

Data available in a publicly accessible repository.

## Supplementary Materials

The following are available online at https://www.mdpi.com/2076-2615/11/1/39/s1, Table S1: Summary of samples information, Table S2: Age, season and reproductive state associated with predominant phylum and alpha diversity based on linear mixed models, Table S3: Differences in the bacterial community composition based on the similarity test of Adonis, Figure S1: Differentiation of gut microbiome structure across age groups.
