# Supplementary material for: Variation of Gut Microbiome in Free-Ranging Female Tibetan Macaques (Macaca thibetana) across Different Reproductive States
Source: Animals (Basel). 2020 Dec 27;11(1):39. doi: 10.3390/ani11010039 (PMC7823274; doi:10.3390/ani11010039)
Supplement: Supplementary file 1 [file animals-11-00039-s001.pdf]

animals

MDPI

Supplementary Material

Table S1. Summary of of sample information.

Animal ID	Samples ID	Age Group*	Reproductive State	Season
HH	3	Middle adult	Lactating	Autumn
HH	25	Middle adult	Lactating	Summer
TH	9	Middle adult	Lactating	Autumn
TH	19	Middle adult	Lactating	Summer
THY	1	Yong adult	Cycling	Autumn
THY	2	Yong adult	Pregnant	Winter
THY	8	Yong adult	Pregnant	Autumn
THY	14	Yong adult	Lactating	Spring
THY	20	Yong adult	Cycling	Summer
TR	12	Middle adult	Cycling	Spring
TR	16	Middle adult	Cycling	Spring
TR	22	Middle adult	Lactating	Summer
TRYu	4	Yong adult	Pregnant	Winter
TRYu	5	Yong adult	Lactating	Autumn
TRYu	11	Yong adult	Pregnant	Spring
TT	10	Old	Lactating	Autumn
TT	17	Old	Cycling	Spring
TXH	7	Yong adult	Lactating	Autumn
TXH	15	Yong adult	Pregnant	Spring
TXH	24	Yong adult	Lactating	Summer
TXX	6	Yong adult	Pregnant	Autumn
TXX	13	Yong adult	Lactating	Spring

TXX	23	Yong adult	Cycling	Summer
YCY	18	Yong adult	Lactating	Summer
YH	21	Middle adult	Lactating	Summer

* Definition of age groups: Yong adult (≥5 ~10 years old), Middle adult (⩾10 ~15 years old), Old (⩾15 years old).

animals MDPI

Table S2. Age, season and reproductive status associated with predominant phylum and alpha diversity based on linear mixed models.

Response variable	Fixed effect				Random effect
	Age (df1, df2; F, P_value)	Reproductive state (df1, df2; F, P_value)	Season (df1, df2; F, P_value)	Reproductive state * Season (df1, df2; F, P_value)	Animal ID (Z, P_value)
Alpha diversity					
ASVs	2, 9; 0.622, 0.558	2, 12; 1.291, 0.312	3, 11; 2.667, 0.098	3, 11; 2.990, 0.076	0.587, 0.557
Shannon	2, 9; 0.045, 0.956	2, 12; 1.134, 0.335	3, 11; 2.500, 0.112	3, 11; 0.651, 0.598	0.468, 0.963
Phylum					
Firmicutes	2, 9; 0.550, 0.595	2, 12; 0.385, 0.688	3, 12; 1.639, 0.233	3, 11; 0.553, 0.657	0.139, 0.889
Bacteroidetes	2, 14; 0.113, 0.894	2, 14; 6.155, **0.012**	3, 14; 3.050, 0.064	3, 14; 0.331, 0.803	NA
Proteobacteria	2, 9; 0.411, 0.5657	2, 12; 5.508, **0.020**	3, 12; 1.254, 0.335	3, 11; 0.049, 0.985	0.401, 0.688
F/B	2, 14; 0.531, 0.599	2, 14; 1.034, 0.381	3; 14; 1.252, 0.329	3, 14; 0.167, 0.917	NA

Note, F/B means the ratio of Firmicutes to Bacteroidetes, Significantly level at the 0.05. NA: not possible to estimate because of the parameter redundancy.

animals MDPI

Table S3. Differences in the bacterial community composition based on the similarity test of Adonis.

Metrics	Treatment	Adonis tests		
		F	R^2	Adjusted p_value
	Cycling vs lactating	1.730	0.092	0.087
Unweigted Unifrac	Cycling vs pregnant	1.150	0.103	0.256
	Lactating vs Pregnant	1.6013	0.086	0.094
Weighted Unifrac	Cycling vs lactating	3.575	0.174	0.003
	Cycling vs pregnant	2.280	0.186	0.014
	Lactating vs Pregnant	0.657	0.0372	0.742

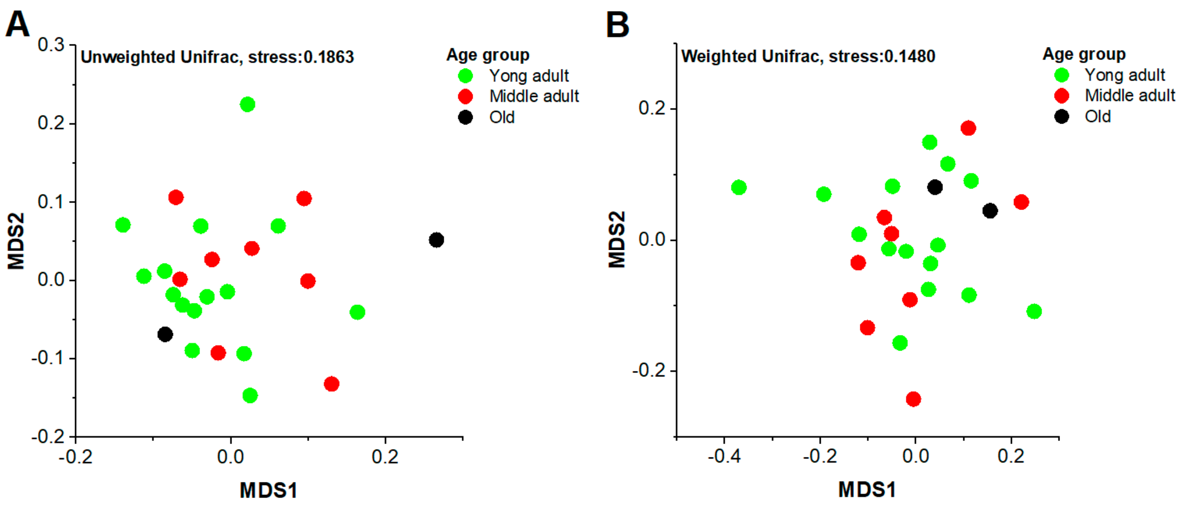

Figure S1. Differentiation of gut microbiome structure across age groups. The analysis is based on the OTU level. NMDS was used to show differentiation patterns of the age groups (A,unweighted; Unifrarc; B, weighted Unifrarc).
